# Selective regulation of nerve growth factor expression in developing cutaneous tissue by early sensory innervation

**DOI:** 10.1186/1749-8104-6-18

**Published:** 2011-04-30

**Authors:** Sean L Wyatt, Bodo Spori, Tom N Vizard, Alun M Davies

**Affiliations:** 1 Molecular Biosciences Research Division, Life Sciences Building, School of Biosciences, Museum Avenue, Cardiff CF10 3AT, UK

## Abstract

**Background:**

In the developing vertebrate peripheral nervous system, the survival of sympathetic neurons and the majority of sensory neurons depends on a supply of nerve growth factor (NGF) from tissues they innervate. Although neurotrophic theory presupposes, and the available evidence suggests, that the level of NGF expression is completely independent of innervation, the possibility that innervation may regulate the timing or level of NGF expression has not been rigorously investigated in a sufficiently well-characterized developing system.

**Results:**

To address this important question, we studied the influence of innervation on the regulation of NGF mRNA expression in the embryonic mouse maxillary process *in vitro *and *in vivo*. The maxillary process receives its innervation from predominantly NGF-dependent sensory neurons of the trigeminal ganglion and is the most densely innervated cutaneous territory with the highest levels of NGF in the embryo. When early, uninnervated maxillary processes were cultured alone, the level of NGF mRNA rose more slowly than in maxillary processes cultured with attached trigeminal ganglia. In contrast to the positive influence of early innervation on NGF mRNA expression, the levels of brain-derived neurotrophic factor (BDNF) mRNA and neurotrophin-3 (NT3) mRNA rose to the same extent in early maxillary processes grown with and without trigeminal ganglia. The level of NGF mRNA, but not BDNF mRNA or NT3 mRNA, was also significantly lower in the maxillary processes of *erbB3*^-/- ^mice, which have substantially fewer trigeminal neurons than wild-type mice.

**Conclusions:**

This selective effect of initial innervation on target field NGF mRNA expression provokes a re-evaluation of a key assertion of neurotrophic theory that the level of NGF expression is independent of innervation.

## Background

Neurotrophic theory provides a widely accepted explanation for how the number of neurons in the developing nervous system is matched to the size and requirements of the targets they innervate. The basic idea is that neurons, which are generated in excess during the early stages of development, depend for their survival on the supply of a neurotrophic factor produced by their targets. The targets produce just the right amount of neurotrophic factor to support the required number of neurons, and superfluous neurons are eliminated in a phase of cell death shortly after the onset of target innervation [1]. Neurotrophic theory is endorsed by a large body of work on nerve growth factor (NGF), the first neurotrophic factor to be identified, and has been corroborated by studies of other members of the NGF family of neurotrophins. Most importantly, NGF has been shown to be produced in the targets of NGF-dependent neurons in proportion to their final innervation density, experimental manipulation of the availability of NGF during development influences the number of NGF-dependent neurons that survive and target-derived NGF is retrogradely transported in endosomes that generate survival signals in cell bodies of the innervating neurons [2-4]. Implicit in the proposal that targets control their innervation density is the assertion that the level of neurotrophic factor synthesis in the target is regulated independently of its innervation. Indeed, the finding in chicken embryos that removal of the neural primordial cells that give rise to neurons that innervate the leg does not prevent NGF mRNA expression in uninnervated leg skin demonstrates that NGF expression can occur independently of innervation [5]. Likewise, the developmental rise in ventricular NGF mRNA is unaffected by sympathectomy part way through this rise [6].

We revisited the issue of the influence of innervation on target field expression of NGF in the most densely innervated cutaneous target with the highest level of NGF synthesis in mouse embryos [7], the maxillary process from which the mystacial whisker pad arises. NGF mRNA expression in the maxillary process commences at embryonic day (E)10.5 and increases with age to peak at E15, with the highest levels in the epithelium [8]. Brain-derived neurotrophic factor (BDNF) mRNA and neurotrophin-3 (NT3) mRNA are detectable in the maxillary process anlagen at E9.5 and peak at E12 and E13, respectively, with the highest levels in the mesenchyme [9]. The earliest axons emerge from the trigeminal ganglion at E9.5 and reach the maxillary epithelium by E11 [10]. The earliest trigeminal neurons depend on NT3 and BDNF for survival, and while some of these neurons soon switch to become NGF-dependent, most NGF-dependent neurons are generated in a wave of neurogenesis between E11.5 and E13.5 [11,12]. With the onset of neuronal loss in the trigeminal ganglion at E13 [10], the great majority of neurons are dependent on NGF for survival [9,13].

## Results

### Influence of innervation on neurotrophin expression *in vitro*

We began investigating the influence of innervation on neurotrophin expression by culturing early maxillary processes with and without attached trigeminal ganglia. These explants were grown in the centre of a Matrigel matrix to maintain their anatomical integrity and were bathed in defined, serum-free medium containing NGF, BDNF and NT3 to maintain optimal neuronal survival. The levels of NGF, BDNF and NT3 mRNAs in the innervated and uninnervated maxillary process explants were quantified using a very sensitive competitive RT-PCR method [14]. The levels of these transcripts are expressed relative to the level of the mRNA for the house-keeping enzyme glyceraldehyde 3-phosphate dehydrogenase (GAPDH) to correct for any small differences in the size of dissected tissue. These cultures were set up at stages when the earliest trigeminal axons reach and begin to innervate the maxillary process *in vivo*, namely, at E10.5 when small numbers of trigeminal axons are starting to grow into the maxillary mesenchyme and at E11 when the first axons come into proximity with the maxillary epithelium [10], where the highest levels of NGF mRNA are expressed later in development [8]. The explants were incubated for 48 hours *in vitro *(equivalent to E12.5 *in vivo *for E10.5 explants and E13 for E11 explants), and thus encompassed the period of development when the levels of NGF, BDNF and NT3 mRNAs are rising rapidly [8,9] as increasing numbers of trigeminal axons reach and innervate the maxillary target *in vivo *[10].

**Figure 1:**
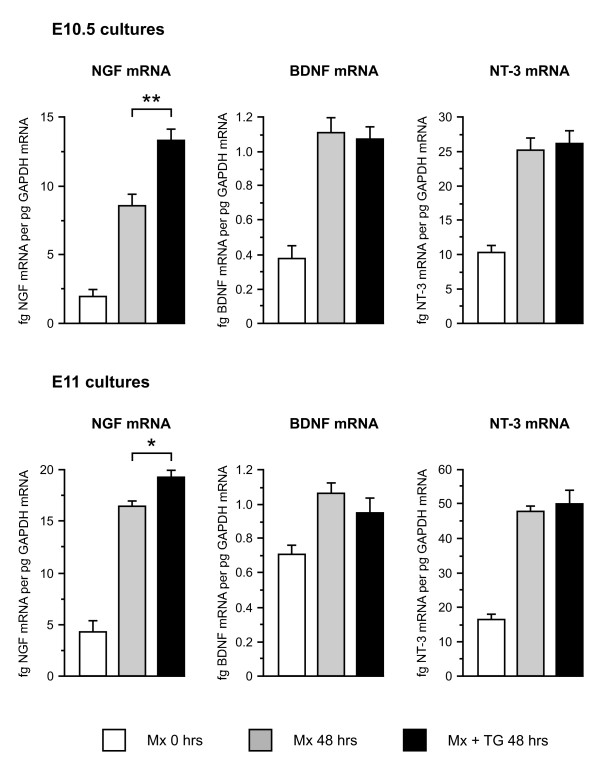
**Levels of NGF, BDNF and NT3 mRNA relative to GAPDH mRNA in maxillary processes dissected from E10.5 and E11 embryos and either not cultured (Mx 0 hours) or cultured for 48 hours alone (Mx 48 hours) or with the trigeminal ganglion attached (Mx + TG 48 hours)**. Statistically significant differences in neurotrophin mRNA levels between maxillary processes cultured for 48 hours alone and maxillary processes cultured with attached trigeminal ganglia were only observed in the case of NGF mRNA: *P < 0.05 (n = 24); **P < 0.001 (n = 23). Means ± standard errors of the mean are shown.

Figure 1 shows that the levels of all three neurotrophin mRNAs increased markedly in E10.5 and E11 maxillary processes cultured for 48 hours relative to the levels in freshly dissected maxillary processes. However, whereas the levels of BDNF and NT3 mRNAs increased to the same extent in maxillary processes grown with and without attached trigeminal ganglia, the levels of NGF mRNA were significantly higher in maxillary processes that retained intact connections with trigeminal ganglia compared with maxillary processes grown alone. The enhanced levels of NGF mRNA in maxillary processes grown with trigeminal ganglia were higher and more significant in E10.5 co-cultures (52% higher, *P *< 0.001, *t*-test, n = 23) than in E11 co-cultures (14% higher, *P *< 0.05, *t*-test, n = 24). The elevated level of NGF mRNA in the maxillary process of co-cultures was unlikely to have been simply due to the trigeminal ganglion improving the viability or increasing growth of maxillary tissue because the levels of BDNF and NT3 mRNAs were no higher in the same maxillary processes (Figure 1).

The selective influence of trigeminal ganglia on NGF mRNA expression could be due to either the presence of increasing numbers of trigeminal axons reaching and innervating the maxillary process from the attached ganglion or to the action of a diffusible agent from the trigeminal ganglion acting on NGF-producing cells within the attached maxillary processes. To investigate whether trigeminal ganglia could affect NGF mRNA expression in early maxillary processes without innervating them, E10.5 maxillary processes and trigeminal ganglia were separated and cultured with a small gap between them. In these co-cultures and in cultures in which single maxillary processes were surrounded by a rosette of six closely apposed trigeminal ganglia and cultured for up to 60 hours, the level of NGF mRNA in maxillary processes co-cultured with trigeminal ganglia was not significantly different from the level of NGF mRNA in maxillary processes grown in isolation (data not shown). This suggests that anatomically appropriate innervation of the maxillary process by trigeminal axons is required for the selective increase in NGF mRNA expression. Taken together, these *in vitro *findings suggest that sensory innervation selectively enhances NGF mRNA expression in the developing maxillary process but does not influence expression of either BDNF mRNA or NT3 mRNA.

### Influence of innervation on neurotrophin expression *in vivo*

**Figure 2:**
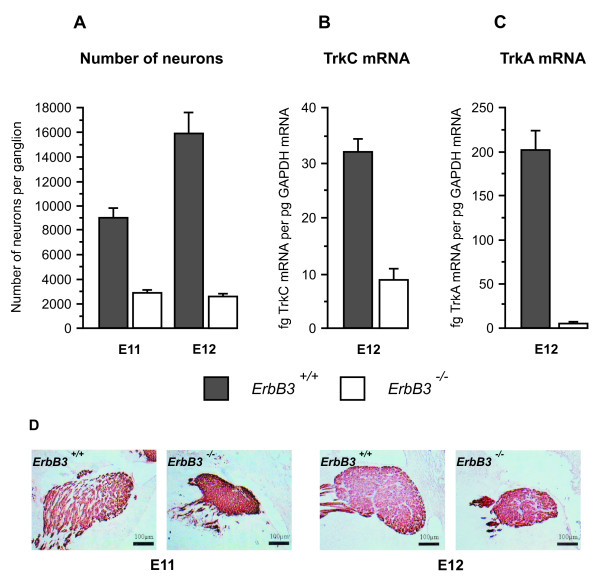
**Loss of neurons and reduced levels of TrkC and TrkA mRNA in the trigeminal ganglia of *erbB3*^-/- ^embryos**. **(A) **Number of neurons in the trigeminal ganglia of *erbB3*^+/+ ^and *erbB3*^-/- ^embryos at E11 and E12. **(B,C) **Levels of TrkC mRNA (B) and TrkA mRNA (C) in the trigeminal ganglia of *erbB3*^+/+ ^and e*rbB3*^-/- ^embryos at E12. Means ± standard error of the mean (SEM) are shown (n = 6 to 8 embryos per data point). **(D) **Sections of the trigeminal ganglia of *erbB3*^+/+ ^and *erbB3*^-/- ^embryos at E11 and E12 labelled with anti-βIII tubulin antibody. Scale bars = 100 μm.

To obtain *in vivo *evidence for the physiological relevance of the above *in vitro *observations, we quantified neurotrophin mRNA levels in the maxillary processes of embryos that have markedly reduced numbers of trigeminal neurons during the early stages of maxillary innervation. Histological analysis of several potentially appropriate transgenic lines revealed that mice deficient in the ErbB3 neuregulin receptor were especially suitable for these studies. *ErbB3*^-/- ^embryos lack Schwann cells and their precursors and lose 82% of lumbar dorsal root ganglia sensory neurons between E12.5 and E18 [15]. In the trigeminal ganglia of *erbB3*^-/- ^embryos we found massive loss of neurons beginning much earlier in development than in dorsal root ganglia, with 67% fewer neurons at E11 and 83% fewer by E12 (Figure 2A). This loss was accompanied by a 73% reduction in the level of TrkC mRNA (Figure 2B) and a 98% reduction in the level of TrkA mRNA (Figure 2C) in the trigeminal ganglia of E12 *erbB3*^-/- ^embryos compared with wild-type littermates. Most TrkA-expressing, NGF-dependent neurons in the trigeminal ganglion are born after TrkB/TrkC-expressing, BDNF/NT3-dependent neurons or are derived from the latter by a developmental switch in Trk expression [9,11-13]. The virtually complete loss of TrkA expression in trigeminal ganglia of *erbB3*^-/- ^embryos by E12 implies the elimination of almost all NGF-responsive trigeminal neurons by this early stage of development.

**Figure 3:**
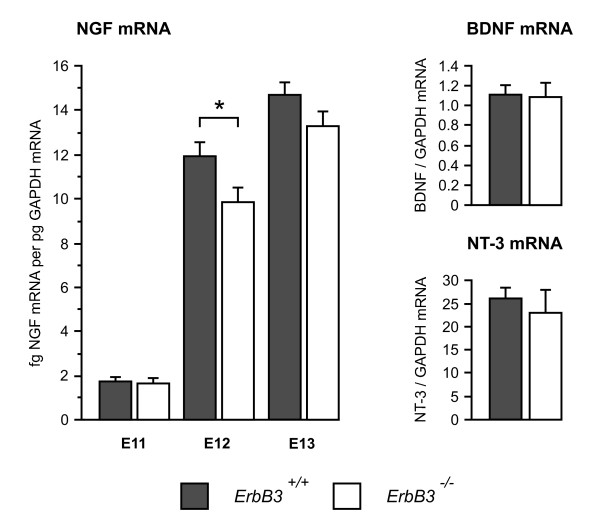
**Levels of NGF mRNA in the maxillary processes of *erbB3*^+/+ ^and *erbB3*^-/- ^embryos at E11 and E12 and levels of BDNF mRNA and NT3 mRNA in the maxillary processes of *erbB3*^+/+ ^and *erbB3*^-/- ^embryos at E12**. A statistically significant difference in neurotrophin mRNA levels between the maxillary processes of *erbB3*^+/+ ^and *erbB3*^-/- ^embryos was only observed for NGF mRNA at E12: *P = 0.015 (n = 34 embryos). Means ± standard errors of the mean are shown.

Figure 3 shows that there were similar levels of NGF mRNA in the maxillary processes of *erbB3*^-/- ^and *erbB3*^+/+ ^embryos at E11, but by E12 there was a highly significant 18% reduction in the level of NGF mRNA in the maxillary processes of *erbB3*^-/- ^embryos compared with wild-type littermates (*P *= 0.015, *t*-test, n = 34 embryos). By E13, although the level of NGF mRNA in the maxillary processes of *erbB3*^-/- ^embryos was just over 9% lower than in wild-type littermates, this difference did not reach statistical significance (*P *= 0.066, *t*-test, n = 28 embryos). In marked contrast to the highly significant differences in the levels of NGF mRNA at E12, the levels of BDNF mRNA and NT3 mRNA were not significantly different in the maxillary processes of *erbB3*^-/- ^and *erbB3*^+/+ ^embryos at this age. There were also no apparent differences in the size or appearance of the maxillary processes of *erbB3*^-/- ^and *erbB3*^+/+ ^embryos. These data suggest that early deficiencies in sensory innervation selectively impair the normal early developmental increase in NGF mRNA expression in the maxillary target *in vivo*.

## Discussion

We have shown that disrupting the early stages of maxillary process innervation by the trigeminal ganglion impedes the developmental increase in NGF mRNA in this cutaneous target field without affecting the magnitude of concomitant developmental increases in the expression of BDNF mRNA and NT3 mRNA. This selective impairment in NGF mRNA expression was most pronounced in maxillary processes cultured in isolation at E10.5 before any sensory axons had reached the cutaneous epithelium, the predominant site of NGF synthesis [8]. Maxillary processes cultured in isolation at E11, after the earliest sensory axons had reached the epithelium [10], still had significantly lower levels of NGF mRNA compared with maxillary processes cultured with attached ganglia, but the relative reduction was less than in isolated E10.5 explants. This suggests that the arrival of the earliest sensory axons in the developing maxillary process selectively enhances NGF mRNA expression. Although not all sensory neurons that innervate the maxillary process are eliminated in *erbB3*^-/- ^embryos, and analysis of the relative levels of TrkC and TrkA mRNA in the early trigeminal ganglia of these embryos suggests that later-generated NGF-responsive neurons are more greatly depleted than early-generated NT3-responsive neurons, there is nonetheless a highly significant reduction in NGF mRNA in the maxillary process of ErbB3-deficient embryos compared with wild-type embryos at E12. Taken together, these *in vitro *and *in vivo *findings suggest that while the timing and level of NGF mRNA expression in the maxillary cutaneous target field is principally controlled by an intrinsic developmental programme operating in this target field, the arrival of sensory axons does selectively enhances NGF mRNA expression. Thus, early developmental changes in the level of NGF mRNA in the maxillary process are not fully independent of innervation.

What developmental role does innervation-dependent NGF regulation play? One possibility is that it contributes to the establishment of regional differences in NGF mRNA expression. In the trigeminal system, the epithelia of the developing maxillary, mandibular and ophthalmic innervation territories express different levels of NGF mRNA at the commencement of the period of naturally occurring neuronal death that are correlated with innervation density [7]. Because the same percentage of neuronal death occurs in the subsets of trigeminal neurons that innervate each of these territories, the regional differences in NGF expression do not sculpt out regional differences in innervation density but maintain them throughout the period of naturally occurring neuronal death [10]. Thus, it is possible the initial regional differences in the numbers of sensory axons reaching these territories may help set the corresponding level of NGF expression in each territory.

An alternative, more plausible role for innervation-dependent NGF regulation is that it helps coordinate the rise in NGF synthesis with the arrival of the initial advancing wave of sensory axons reaching the target field. This idea is consistent with the developmental lag in NGF mRNA expression in *erbB3*^-/- ^embryos compared to wild-type littermates. When the first few axons reach the maxillary epithelium at E11, there is no difference in the low levels of NGF mRNA in the maxillary processes of *erbB3*^-/- ^and *erbB3*^+/+ ^embryos, but by E12 when large numbers of axons are beginning to arrive in the maxillary process in normal development, there is a highly significant reduction in the level of NGF mRNA in ErbB3-deficient embryos. However, by E13, the level of NGF mRNA in the maxillary process of *erbB3*^-/- ^embryos has begun to catch up with the level in *erbB3*^+/+ ^embryos. Thus, these findings would appear to reveal the operation of a mechanism that helps coordinate the timing of trophic interactions between neurons and their targets during development. A similar mechanism has been proposed for coordinating the onset of neurotrophin dependence in certain populations of sensory neurons with the arrival of their axons at their targets [16]. In nodose ganglion neurons, whose axons have a comparatively long distance to grow to reach their targets and have a correspondingly protracted period of neurotrophin-independent survival before they become dependent on BDNF for survival [16,17], the onset of BDNF dependence can be accelerated in culture by briefly exposing the neurons to BDNF toward the end of the period of neurotrophin independence [16]. Thus, neurotrophin-promoted acquisition of neurotrophin dependence and innervation-dependent regulation of target field expression of NGF may, in certain neuronal systems, contribute to the temporal coordination of trophic interactions between neurons and their targets during development.

Our finding that the developmental increase in NT3 mRNA in the maxillary process is independent of innervation agrees with the demonstration that NT3 mRNA expression in the limb bud is also independent of sensory innervation [18]. However, our demonstration that early sensory innervation enhances NGF mRNA expression in the maxillary process conflicts with the conclusion, based on studies of the consequences of disrupting innervation to the developing limb bud and heart, that the level of NGF mRNA expression in the targets of NGF-dependent neurons is independent of innervation [5,6]. While modulation of NGF mRNA expression by innervation may not be a universal feature of the target fields of all NGF-dependent populations of neurons, it is possible that the very high levels of NGF mRNA and dense innervation of the maxillary process together with the very sensitive competitive RT-PCR method used in our study have facilitated detection of the effects of early innervation on NGF mRNA expression. Furthermore, if NGF mRNA expression is modulated primarily by the earliest wave of axons to reach the target field as part of a mechanism to coordinate the onset of NGF synthesis with the onset of innervation, it would be necessary to disrupt the very earliest stages of target field innervation and study the consequences of such disruption during these same early stages in order to observe an effect on NGF mRNA expression. In the study of chicken embryo limb bud innervation, while successful removal of the neural primordial cells that give rise to the neurons that innervate the limb would have eliminated innervation from the outset, the consequences for NGF mRNA expression were not first studied until 3 days after the earliest sensory axons would have normally reached their cutaneous targets [5], with the possibility that any early effect on NGF mRNA expression might have been missed. In the study of postnatal rat heart innervation, chemical sympathectomy did not commence until 5 days after the earliest sympathetic axons had reached the targets [6], which might have been too late to observe an early effect of the arrival of sympathetic axons on target field expression of NGF.

The cellular and molecular mechanisms by which early sensory innervation selectively influences NGF expression in the cutaneous target field of the developing maxillary process are important unanswered questions. Whether this requires direct cell-cell contact and signalling between the arriving axons and the NGF-producing cells in this target field or whether it depends on the synthesis and release of some signalling molecule by the early axons remains to be determined. It is also feasible that the Schwann cell precursors that accompany the early axons into the target field might provide the signal that modulates NGF expression. It will also be important to determine whether the level of expression of NGF and possibly other neurotrophins is also partly regulated by early innervation elsewhere in the embryo. Finally, to provide an understanding of the developmental significance of the fine temporal modulation of NGF expression by early innervation, it will be instructive to interfere with the underlying signalling mechanism that mediates this and study the consequences for the regulation of neuronal survival and the patterning of innervation.

## Conclusions

Our findings provoke a reassessment of a key tenet of neurotrophic theory, the assertion that neurotrophic factor expression in the target is regulated completely independently of its innervation. Our demonstration of the selective enhancement of NGF mRNA expression in the maxillary process by early sensory innervation suggests the operation of a mechanism that helps coordinate the timing of trophic interactions between neurons and their targets. This may be important to ensure NGF is produced at the right time to sustain the survival of the earliest neurons to innervate their targets.

## Materials and methods

### Cultures

Maxillary processes alone or with the adjoining tissue that contains the entire trigeminal ganglion were dissected from E10.5 and E11 mouse embryos (Theiler stages 17 and 18, respectively) obtained from overnight matings of CD1 mice. Each explant was cultured separately in a single well of Nunclon 4-well dishes in a 40 μl Matrigel matrix (BD Biosciences, Oxford, Oxfordshire, UK with 0.5 ml of defined medium [19] supplemented with 10 ng/ml NGF, 10 ng/ml BDNF and 10 ng/ml NT3. These neurotrophins were included to ensure optimal neuronal survival in explants containing trigeminal ganglia, but were also included in the medium used for culturing maxillary processes on their own to keep culture conditions the same.

### Measurement of mRNA levels

NGF, BDNF, NT3, TrkA, TrkC and GAPDH mRNA levels were measured in total RNA extracted from maxillary processes or trigeminal ganglia by competitive RT-PCR [14]. Briefly, known amounts of cRNA transcripts representing NGF, BDNF, NT3, TrkA, TrkC and GAPDH coding sequences were added to reverse transcription reactions that contained total RNA extracted from maxillary processes. Reverse transcription reactions were performed using Superscript reverse transcriptase (Invitrogen Paisley, Renfrewshire, UK according to the manufacturers' protocol. The cRNA transcripts of the genes were transcribed *in vitro *from a corresponding cloned cDNA copy of the genes that had been modified to add four base pairs in between the forward and reverse PCR assay primers. PCR amplification of cDNA was performed using Helena Biosciences Taq and gene-specific primers according to the Taq manufacturers' protocol (Helena Biosciences, Gateshead, Tyne and Wear, UK). Following PCR, the RT-PCR products of the endogenous mRNA and the competitor cRNA were separated on 8% polyacrylamide gels and stained with SYBR Gold Molecular Probes (Invitrogen Paisley, Renfrewshire, UK. Images of stained gels were captured on a gel documentation apparatus and image analysis software was used to determine the ratio between the RT-PCR products of the endogenous mRNA and the competitor cRNA, therefore allowing quantification of mRNA levels in the original RNA sample. The primer sequences were: NT3 forward, 5'-TACTACGGCAACAGAGACG-3'; NT3 reverse, 5'-GTTGCCCACATAATCCTCC-3'; NGF forward, 5'-AGCATTCCCTTGACACAG-3'; NGF reverse, 5'-GGTCTACAGTGATGTTGC-3'; BDNF forward, 5'-ACTTGGCCTACCCAGGTGTG-3'; BDNF reverse, 5'-TGTCGTCGTCAGACCTCTCG-3'; TrkA forward, 5'-CGTCATGGCTGCTTTTATGG-3'; TrkA reverse, 5'-ACTGGCGAGAAGGAGACAG-3'; TrkC forward, 5'-CCCACCAAAGACAAGATG-3'; TrkC reverse, TATCCAGTCCACATCAGG-3'; GAPDH forward, 5'-TCCAGTATGACTCCACTCAC-3'; GAPDH reverse, 5'-TCCTGGAAGATGGTGATGG-3'. Annealing temperatures for the NT3, NGF, BDNF, TrkA, TrkC and GAPDH primers were 55°C, 50°C, 60°C 56°C, 52°C and 51°C, respectively.

### Quantification of the number of neurons in the trigeminal ganglia

The heads from E11 and E12 *ErbB3*-/- and wild-type littermates were fixed for 5 and 15 minutes, respectively, in Carnoy's fluid (60% ethanol, 30% chloroform, and 10% glacial acetic acid). Following dehydration through a graded alcohol series, the tissue was paraffin wax embedded. Serial sections of the heads were cut at 8 μm and were mounted onto poly-lysine-coated slides (VWR, Radnor, Pennsylvania, USA or Gold Seal Ultrastick Slides (Erie Scientific, Portsmouth, New Hampshire, USA. To identify all neurons in these sections, the sections were stained for βIII-tubulin. Sections were cleared in xylene and rehydrated prior to quenching in 0.3% hydrogen peroxide and 10% methanol in PBS for 20 minutes. Non-specific antibody binding was blocked in 10% horse serum, 0.4% Triton X-100 in PBS prior to incubation with mouse anti-βIII tubulin antibody (Promega, Madison, Wisconsin, USA diluted 1:2,000 in PBS overnight at 4°C. The cells were then labelled using biotinylated secondary antibody (1:200), avidin, biotinylated horseradish peroxidase macromolecular complex (Vectastain ABC Kit, Vector Labs, Burlingame, California, USA. The substrate used for the reaction was 1 mg/ml diaminobenzidine tetrachloride (FastDAB, Sigma-Aldrich, St Louis, Missouri, USA. Neuronal number was quantified using a digital stereology system that employs a combination of the optical dissector and volume fraction/Cavalieri methods (Kinetics Imaging, Ltd., Bromborough, Merseyside, UK.

## Abbreviations

BDNF: brain-derived neurotrophic factor; E: embryonic day; GAPDH: glyceraldehyde 3-phosphate dehydrogenase; NGF: nerve growth factor; NT3: neurotrophin-3; PBS: phosphate-buffered saline; Trk: tyrosine receptor kinase.

## Competing interests

The authors declare that they have no competing interests.

## Authors' contributions

SLW designed the very sensitive RNA quantification strategy and carried out most of this quantitative work, BS carried most of the cell culture and histology, TNV contributed to the histology and illustrations, and AMD conceived the study and wrote the paper. All authors read and approved the final manuscript.
